# Refining Accelerometer‐Based Animal Behaviour Classifications With Sequence‐Informed Post‐Processing

**DOI:** 10.1002/ece3.74053

**Published:** 2026-08-02

**Authors:** Oakleigh Wilson, Hui Yu, David Schoeman, Gabriella Sparkes, Christofer Clemente

**Affiliations:** ^1^ School of Science, Technology and Engineering University of the Sunshine Coast Sippy Downs Queensland Australia; ^2^ School of Life and Environmental Sciences Deakin University Waurn Ponds Victoria Australia; ^3^ School of the Environment University of Queensland Brisbane Queensland Australia

**Keywords:** behaviour, biologging, classification, machine learning, post‐processing

## Abstract

Supervised machine learning has been used to detect fine‐scale behaviours from animal‐borne accelerometers by dividing the continuous sequences of behaviour into discrete segments and classifying each with a distinct behavioural category. This approach, while widely implemented, discards the sequential information available in the temporal ordering of the behavioural series. ‘Post‐processing’ (smoothing and error correction made after the initial classifications) can be used to improve the accuracy of the original predictions by learning from the natural transitions and durations of behaviours. While broadly implemented across other classification domains, this technique has been underutilised for accelerometer‐based animal behaviour classification. In this paper, we compare the performance of five different post‐processors (modal, duration‐based, transition‐based, Hidden Markov Model, and a Naive Bayes smoother) against the original predictions from the base classifier across 15 animal accelerometer datasets across 13 species. Overall, while there was no single post‐processing method that was optimal in every dataset, we find Bayesian smoothing to have the best overall performance improvement (average 6.4% increase in *F*1‐score), resulting in ecological interpretation closest to the true data. Requiring no additional data and very little additional computational effort, this preliminary research suggests promising potential for the widespread and accessible inclusion of post‐processing in the animal behaviour classification pipeline.

## Graphical abstract

Machine learning can be used to detect fine‐scale animal behaviours from accelerometer data but even the best model will make prediction errors. Logic‐based post‐processing techniques can be used to correct for those errors, with no additional data or computation. We demonstrate up to 20% performance improvement using post‐processing, as well as highlighting the ecological importance of smoothing incorrect predictions.

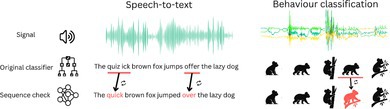

## 1 Introduction

Machine‐learning (ML) models can be trained to detect specific fine‐scale behaviours from animal‐borne accelerometer data. Despite growing sophistication in model design, however, even the best behavioural models face some fundamental constraints. Limitations in collections of field data mean some behaviours are not observed sufficiently to be included in the classification models, while others (due to rarity, context dependence, or high variability) are difficult to classify with confidence (Sur et al. 2017; Aulsebrook et al. 2024). Even for models trained on datasets with complete behavioural repertoires, predictive performance nearly always declines when the model is applied on data from new individuals (Ferdinandy et al. 2020; Rast et al. 2020; Dickinson et al. 2021). Thus, while the majority of methodological research has focused on improving model performance through algorithm tuning, additional sensor axes, or novel feature sets (e.g., Chandrashekar and Sahin 2014; Kate et al. 2016; Ladds et al. 2016), it is improbable that perfect classification will ever be achieved. Given the inevitability of imperfect classification, therefore, ecologists are well‐served considering techniques for managing and interpreting this residual error.

**FIGURE 1:**
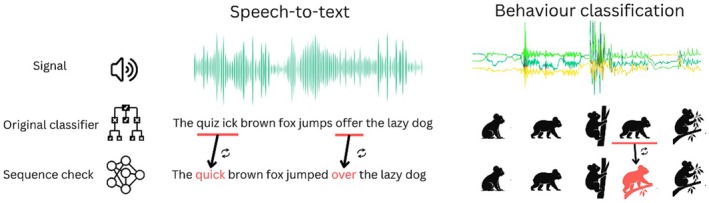
Conceptual examples of post‐processing. A first classification ML model detects specific events in the signal (words from speech, left, and behaviours from acceleration trace, right). A secondary model flags unlikely event progressions and changes these to more probable events (grammar checker, left, behavioural sequence checker, right). In the speech example (left), the misclassified words are replaced with a similar‐sounding but semantically more meaningful substitute. In the behavioural example (right), the misclassified behavioural event is replaced with a similar event, but in the correct sequential context (i.e., after climbing a tree, a koala cannot be walking on the ground, therefore the event is substituted with the contextually more likely event of walking on a branch).

In other fields of ML, it is standard practice to manage model prediction error through post‐processing. Post‐processing comprises all forms of secondary inference that refines model prediction. Typically, this involves applying contextual knowledge and domain‐specific constraints via additional models or rule‐based logic to adjust or smooth the ML outputs, correcting initial misclassifications. This approach is routinely implemented across a range of fields. In speech recognition, grammar rules are used to flag and correct implausible word sequences (Mehrish et al. 2023) (Figure 1); in genetics, post‐processing is used during DNA sequence recombination to prevent duplication and deletion (Borges et al. 2020); in medical diagnostics, predictions are aligned with known symptom–disease relationships to limit implausible or misleading diagnoses (Turbé et al. 2023); and in weather forecasts, adjacent locations' predictions are combined and reconciled to smooth differences (Hewson and Pillosu 2021). In these cases, the ML model prediction is seen as only one step in a broader interpretive process. Even in non‐ML fields such as GPS‐based animal location estimation, post‐processing is routinely implemented to remove noise and misreads (Saldanha et al. 2023).

In contrast, post‐processing remains largely absent from animal accelerometry pipelines. Classified behavioural labels are treated as definitive categories, rather than as probabilistic predictions (outside of threshold‐based classification e.g., Glass et al. 2020; Rast et al. 2020) with few studies attempting to correct or refine model outputs after prediction (except see: Grünewälder et al. 2012; Yu and Klaassen 2022; Resheff et al. 2022). Recently, however, the use of temporal‐context averaging windows to smooth probabilities across behavioural class transitions and remove improbable behaviours has been shown to result in more reliable classifications (Agarwal et al. 2026) suggesting the utility of the sequence‐based post‐processing approach in animal accelerometry.

Behavioural data are sequentially dependent time‐series data, such that the current state depends on states in the past at scales of seconds, minutes, and longer‐term daily, seasonal, and annual intervals (Leos‐Barajas, Gangloff, et al. 2017; Minasandra et al. 2025). Standard accelerometer analysis pipelines, however, implementing statistical‐based ML models (as opposed to deep‐learning models which intake raw data) require division of continuous behavioural sequences into discrete windows to generate summary features (Barwick et al. 2020). These windows are then shuffled and treated as independent data points for both training and prediction, discarding useful information that could help correct misclassifications. This sequential dependency has been considered by some researchers when producing initial classifications, such as by implementing sequence‐based ML classifiers, e.g., Hidden Markov Models (HMM; Leos‐Barajas, Photopoulou, et al. 2017; Conners et al. 2021) and Long Short‐Term Memory Models (LSTM; Hussain et al. 2022; Otsuka et al. 2024). However, the majority of the accelerometer‐based behaviour‐classification literature analyses accelerometer data without the context of time and sequence.

The literature that has explored the possibility of post‐processing has demonstrated the potential of several methods to achieve performance improvements. Known error in the confusion matrix has been used to correct for biases in overall time‐budgets (Resheff et al. 2022), though this does not apply to individual classifications. Post‐processing for reclassification of individual events has been demonstrated through the application of a Hidden Markov Model (HMM) to smooth time dependencies after initial classification (Grünewälder et al. 2012), duration‐based logic‐gates to eliminate behaviours with improbably short durations (Yu and Klaassen 2022), and averaging of neighbouring probabilities to smooth out improbable transitions (Agarwal et al. 2026). However, thus far, these techniques have been demonstrated only in isolation on singular datasets, with the generalisation of these techniques not yet assessed.

Here, we argue that post‐processing represents a critical and underutilised stage in the interpretation of accelerometer‐derived behavioural classifications and that it has potential as a pragmatic and accessible path to increase interpretability of behaviour predictions while decreasing the burden of assuming perfect prediction in the ML stage. By integrating this knowledge into the generalised post‐prediction workflow, we could correct misclassifications, fill gaps, and improve the ecological plausibility of behavioural classifications without needing perfect accuracy from the classification model itself. We hypothesise that post‐processing can substantially enhance the accuracy of behaviour classification, relative to the model's unprocessed predictions. Specifically, we predict that datasets where the training data contains a high number of natural behavioural transitions will benefit most from post‐processing.

## 2 Methods

### 2.1 Data

Data for this study was raw animal‐borne accelerometer data, including sampling timestamps, ID of sampled individual, and unprocessed tri‐axial accelerometer recordings. Data was acquired from 15 sources, capturing raw accelerometer movement data from 13 species. Datasets sources and characteristics are described in Table 1 below. Datasets were selected to represent the range of accelerometer datasets currently in the literature, with a range of sampling rates (1–100 Hz), body masses (0.5–600 kg), and morphologies (marine, terrestrial, and avian). Labelled data contained ground‐truthed behavioural labels and was used as the training dataset. Labelled data was collected in a variety of ways, as outlined in each of the associated papers. The most common method, however, was to observe multiple individuals with the biologging accelerometer during captivity over several days and later time‐synchronise observations with the accelerometer signal (e.g., Annett et al. 2023; Sparkes et al. 2025). In all but one dataset, behaviours were expressed freely, whether in the wild or captive enclosure, and recorded as such. In the Vehkaoja_Dog dataset, however, behaviours were expressed as instructed by the human companion.

**TABLE 1:** Sources and characteristics of each of the animal‐borne accelerometer datasets included in the study.

Dataset key	Species	Paper and dataset citation	Individuals	Included behaviours	Sample rate (Hz)	Total transitions	Mean transitions per continuous sequence	Proportion of sequences with transitions	Mean continuous annotated sequence length (sec)
Clemente_Echidna	Short‐beaked Echidna	Clemente et al. (2016) Personal correspondence	8	4	10	665	82.12	1	67111.75
Desantis_Rattlesnake	Rattlesnake	DeSantis et al. (2020), Hoffman et al. (2023)	13	2	1	215	0.42	0.13	755.03
Dunford_Cat	Domestic Cat	Dunford et al. (2024), Dunford (2024)	9	7	40	309	2.09	0.32	32.50
Galea_Cat	Domestic Cat	Galea et al. (2021) Personal correspondence	12	6	50	277	0.28	0.13	29.47
HarveyCaroll_Pangolin	African Ground Pangolin	Harvey‐Carroll et al. (2024), Carroll and Harvey‐Carroll (2024)	10	6	50	725	0.44	0.22	15.67
Jeantet_Turtle	Green Turtle	Jeantet et al. (2020a, 2020b)	6	8	20	9161	0.29	0.13	35.08
Ladds_Seal	Australian fur seals, New Zealand fur seals, subantarctic fur seal, and Australian sea lions	Ladds et al. (2016), Ladds (2021)	12	4	25	3028	3.91	0.53	62.65
Maekawa_Gull	Black‐tailed gulls	Korpela et al. (2020), Hoffman et al. (2023)	11	4	25	175	0.88	0.26	3605.82
Mauny_Goat	Dairy Alpine Goat	Mauny et al. (2024, 2025)	8	5	5	324	0.02	0.02	149.25
Pagano_Bear	Polar Bear and Brown Bear	Pagano et al. (2017), Hoffman et al. (2023)	6	7	16	5356	0.72	0.15	15.76
Smit_Cat	Domestic Cat	Smit et al. (2023) Personal correspondence	6	7	30	2269	3.47	0.67	130.73
Sparkes_Koala	Koala	Sparkes et al. (2025) Personal correspondence	11	5	50	350	0.4	0.06	18.03
Studd_Squirrel	Yukon Red Squirrel	Studd et al. (2018a, 2018b)	29	5	1	1411	1.82	0.56	123.23
Vehkaoja_Dog	Domestic dog	Vehkaoja et al. (2022a, 2022b)	62	10	100	5399	1.92	0.31	37.35
Yu_Duck	Pacific black duck	Yu et al. (2022), Yu and Klaassen (2022)	7	10	25	436	2.33	0.51	73.08

The Biologger Ethogram Benchmark (BEBE; Hoffman et al. 2024) is a pre‐existing collection of collated animal‐borne accelerometer datasets intended to be used for comparative classification tasks exactly as undertaken in this study. However, the data included in the BEBE do not always include the timestamp of the behavioural event. As this present study required identification of continuous vs. non‐continuous sequences, non‐timestamped datasets could not be used. 6 BEBE datasets were incorporated, with an additional 9 datasets gathered from primary sources.

For each dataset, metrics of the ‘continuousness’ of records in the training data were collected—i.e., how much of the training data was annotated in continuous sequence, containing natural transitions between behaviours, as opposed to curated and cleaned training data with a single behaviour per continuous sampling. To calculate this, all continuous sequences of data were extracted and the number of transitions (changes in behavioural label) present in the data was recorded. For each dataset, the following metrics were calculated:

- Proportion including transitions: of all continuous sequences, how many contained behavioural transitions. Expressed as a decimal percentage.
- Average transitions per sequence: across all continuous sequences, what was the average number of behavioural transitions recorded? Expressed as numeric count.

### 2.2 Producing Predictions

#### 2.2.1 Feature Generation

All raw data was processed into features within non‐overlapping windows (i.e., contiguous segments of data, with no data shared between segments) of 2 s for all datasets except Studd_Squirrel and Desantis_Rattlesnake. These datasets had a sampling frequency of 1 Hz and were processed to 5‐s windows to ensure sufficient samples to generate time‐series features. For each window, a total of 203 features were generated, including the features specified in Tatler et al. (2018) and all time series features from the R package ‘tsfeatures’ (Hyndman et al. 2023). Time was set as the first increment of that window. In the labelled data, ‘Activity’ was set as the most common label for that window.

#### 2.2.2 Model Tuning, Training, and Testing

To achieve cross‐validation, the entire process of tuning and training the ML model, generating the predictions, and trialling each post‐processing approach was replicated on three folds of the data. Test sets were defined by randomly assigning individuals equally to 3 folds. In each round, a different one‐third of individuals served as the test set and the remaining two‐thirds as training such that all individuals served in the test set once each. The remainder of the non‐test data per fold was used to tune and train the behavioural classification model (where it was iteratively split into training and validation sets).

The base behavioural classification model architecture was the Random Forest (RF) from the ‘ranger’ package. The RF is commonly used across accelerometer literature (e.g., Studd et al. 2018a; Pagano et al. 2017; Tatler et al. 2018). Tuning of the RF involves adjusting the hyperparameters of the model (parameters controlling model shape and learning ability that are set prior to learning from the data) to customise it to the data and problem. The RF model has hyperparameters for the number of trees in the forest (*number.trees*, trialled 10–1000), the maximum number of variables to be considered at each node (*mtry*, trialled 3–20), and the depth of the trees (*max.depth*, trialled 10–50). The optimal value of these hyperparameters was tuned using the ‘rBayesianOptimisation’ (Yan 2025) package.

For each set of possible hyperparameters, within the cross‐validation fold, the model was trained and tested three times using individual‐based bootstrapping (repeated resampling, stratified by individual) such that 80% of individuals' datasets were used as training data and the remaining 20% were used as the validation set, but for each repetition, the individuals in each set were randomised. Due to class imbalance, models were weighted by the prevalence of each class in the training data. Per‐class performance was measured using the *F*1‐score, a compound metric robust to class imbalances (Sokolova and Lapalme 2009; Chicco 2017). *F*1‐score was calculated as:

$$\begin{array}{c} F1-\text{score}=\text{harmonic mean of precision and recall}\\ =2\times \left(\text{Precision}\times \text{Recall}\right)/\left(\text{Precision}+\text{Recall}\right) \end{array}$$

where Precision and Recall are calculated as follows:

$$\begin{array}{cc} \text{Precision} & =\text{samples correctly classified}\;\mathrm{as}\;\text{positive}/\mathrm{all}\;\text{samples classified}\;\mathrm{as}\;\text{positive}\\ & =\text{True Positive}/\left(\text{True Positive}+\text{False Positive}\right) \end{array}$$

$$\begin{array}{cc} \text{Recall} & =\text{samples correctly classified}\;\mathrm{as}\;\text{positive}/\mathrm{all}\;\text{samples truly positive}\\ & =\text{True Positive}/\left(\text{True Positive}+\text{False Negative}\right) \end{array}$$

Overall performance was calculated as the macro‐average *F*1‐score (i.e., average *F*1‐score of individual classes, equally weighted irrespective of their prevalence in the validation data [Sokolova and Lapalme 2009]), further averaged across the three replicates. The hyperparameter set with the highest overall average macro‐averaged *F*1‐score was selected as the optimal model for that dataset. These optimal hyperparameters were used to train a final model including all non‐test data.

#### 2.2.3 Model Prediction

The final model was used to predict behavioural classes onto the hold‐out test data. Each prediction was expressed as the probability that the event belonged to each of the possible classes, and the ‘Activity’ prediction was selected as the class with the greatest probability. Additionally, the models were applied back to the training data to provide a ‘training example’ of error in the model (see below for further description).

### 2.3 Post‐Processing

#### 2.3.1 Applying Smoothing Algorithms

To determine the effect of post‐processing on the classification output of the models, five potential post‐processing methods were compared to the control (no post‐processing) predictions. These methods were as follows: in Table 2 (more details provided in the linked github).

**TABLE 2:** Summarisation of five post‐processing methods trialled in this analysis.

Method	Description	Link
None (control)	No post‐processing was applied	
Mode	A rolling window of five consecutive predictions was applied. The central prediction in each window was replaced with the modal (most frequent) behaviour within the rolling window	Code for Mode method
Duration	The 5th percentile of the observed durations for each behaviour was calculated from the ground‐truthed training data (shorter than 95% of observed events in that behavioural class). In the test data, any predicted sequence shorter than this threshold was coerced to the sequentially preceding behavioural class to eliminate implausibly brief events	Code for Duration method
Transition	A transition probability matrix was calculated from the training data, capturing the likelihood of one behaviour transitioning to another within each continuous recording sample. In the test data, transitions with less than twice chance rate probability (i.e., less than twice the rate of probability that any class would be selected by chance) were considered biologically implausible and corrected by reverting the transition to the preceding behavioural class	Code for Transition method
HMM	A Hidden Markov Model was trained on the training data with the ground‐truthed labels used as the true states and the predictions as the emissions. This model was then applied to the test data predictions (emissions) to determine the ‘true states’ as smoothed predictions within each continuous recording sample. HMM explained in detail: (Ruiz‐Suarez et al. 2022)	Code for HMM method
Bayesian	Bayesian smoothing was applied by updating each prediction by incorporating prior behavioural classes, the probability of transition to the current behaviour, as well as the likelihood of observing a given prediction given the true class (as learnt from the training data). Bayesian smoothing explained in detail: (van de Schoot et al. 2021)	Code for Bayesian method

Each method was applied to predictions from each species' test data to produce post‐processing corrected ‘smoothed predictions’.

#### 2.3.2 Performance of Smoothing Algorithms

Performance was calculated in terms of improvement to classification performance by calculating both class‐specific as well as macro‐averaged Precision, Recall, and *F*1‐score, averaged across the three cross‐validation folds (with non‐predicted classes set to *F*1 of 0 to penalise the model for failing to predict classes). The model with the greatest average increase in *F*1 compared to the control (no smoothing) was considered the optimal post‐processing method for that dataset. The difference between the average *F*1‐score for each post‐processing method and the score for the control method was calculated as the ‘relative improvement’ (improvement/original *F*1‐score). A mixed‐effects model was constructed with relative improvement as the dependent variable (global intercept as 0 to compare to a null hypothesis of no change), with smoothing method, proportion of sequences including transitions, average transitions per sequence, and number of sequences that contained multiple behaviours as predictor variables, and species and replicates within species as random effects. Model fit was assessed using Akaike Information Criterion (AIC) (Goodfellow et al. 2016).

To specifically investigate effects of the post‐processing approaches on the minority classes, each behavioural class was defined based on prevalence. Considering equal prevalence as the expected prevalence if all classes were equally represented, minority classes were those with less than their expected prevalence, majority classes were those with more than double their expected prevalence, and balanced classes were those remaining. ‘Relative improvement’ was calculated for each subset of class prevalence based on the *F*1‐score as well as Recall and Precision.

### 2.4 Ecological Sequence Case‐Study

To assess the downstream analysis and ecological interpretation implications of post‐processing, a case study was conducted using data from the Sparkes_Koala dataset (Sparkes et al. 2025; data provided by personal correspondence). Rather than just addressing the accuracy of the model, the performance was assessed in terms of an ecological question—the frequency and duration of locomotion events (i.e., walking on the ground). As well as the labelled data, an additional unlabelled dataset was collected from tracked koalas in the wild. Continuous unlabelled raw data (12 h; 4 pm–4 am beginning on the second afternoon of deployment) was selected from the experimental deployment period from each of five individuals. For each individual, features were generated according to the protocol above and behavioural predictions generated from the optimal ML model (as determined in the hyperparameter optimisation steps earlier) and trained on all available labelled data. These raw predictions were smoothed with the five post‐processors developed above. As the true behavioural class for the deployment period is not known, the classification performance for these predictions cannot be assessed. Instead, a difference in the answer to the ecological question—in this case, the frequency and duration of locomotion events—was assessed between the control and five post‐processing methods.

All analysis undertaken in R, version 4.4.2. All code available at https://github.com/OakAlice/PostProcessing.

## 3 Results

### 3.1 Performance Changes

**FIGURE 2:**
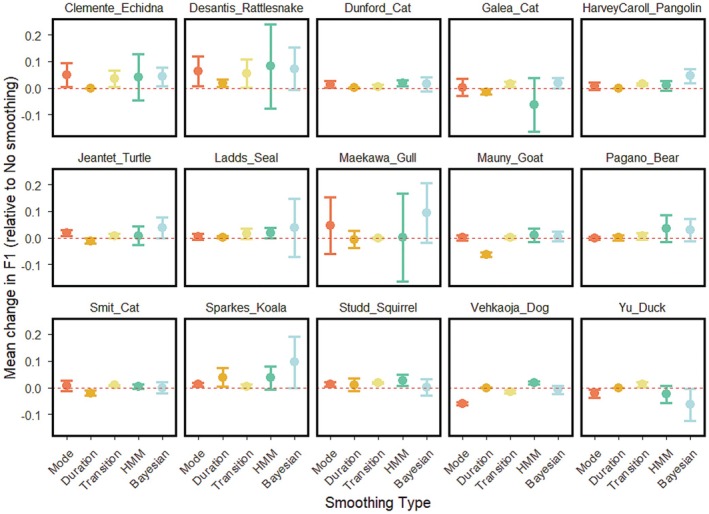
Relative percentage change in macro‐average *F*1‐score for post‐processing methods for each of the individual datasets where change is calculated relative to the control method as (smoothing score—baseline score)/baseline score. The horizontal line represents no change (baseline control, no post‐processing). Points represent mean with error bars for 95% confidence intervals.

The performance of the base classifier with no post‐processing (control) differed between species with a range 0.79 (DeSantis_rattlesnake, 2 classes) to 0.23 (Mauny_Goat, 5 classes) macro‐average *F*1‐score (mean performance 0.47). Changes to *F*1 performance due to post‐processing ranged from −6 to 9 *F*1‐score depending on the post‐processing method. Expressed as a percentage performance change relative to the initial performance for each dataset, effects ranged between −27% and 20% change to the *F*1‐score (Figure 2).

**FIGURE 3:**
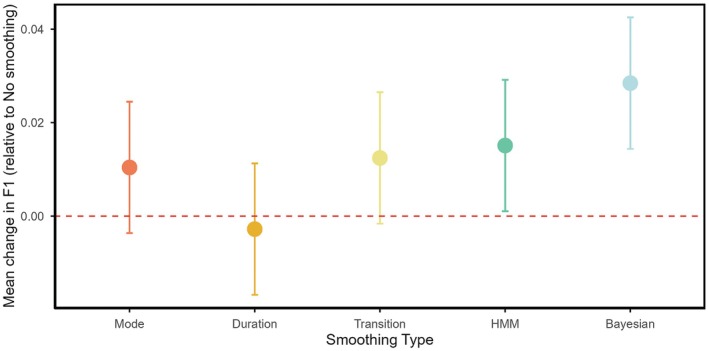
Change in macro‐average *F*1‐score for post‐processing methods (averaged over random effects) where change is calculated relative to the control method. The horizontal line represents no change (baseline control, no post‐processing). Points represent mean with error bars for 95% confidence intervals.

All datasets had at least one post‐processing method that resulted in improved overall *F*1‐score performance, and all methods resulted in a positive improvement in at least one dataset. A linear mixed‐effects model was used to assess the effects of the post‐processing method on the change in classification performance, with Species and cross‐validation replications included as random effects and no intercept (thus, all compared to 0—control, no post‐processing) (Figure 3). Bayesian smoothing had a significant positive effect on global change to the *F*1‐score (*β* = 0.028, *p* < 0.001), resulting in a relative increase of 6.4% across all datasets with a maximum increase of 9 *F*1‐score (relative increase of 20% and 13% in the Maekawa_Gull and Sparkes_Koala datasets, respectively).

Additionally, the HMM had a significant positive effect on global relative *F*1‐score (*β* = 0.015, *p* < 0.05), but a mean improvement of 1.5 *F*1‐score (mean percentage relative change 4.1%) across all datasets. Other post‐processing methods did not statistically significantly differ from 0, though Transition had marginal positive effects (*β* = 0.012, *p* = 0.083).

To explore the variance in performance among methods, several covariates were assessed, including total number of behavioural transitions captured in the training data, proportion of continuous sampling sequences that contained a behavioural transition, mean number of transitions captured in any continuous sampling sequences, and total number of behavioural classes. A full model was constructed to quantify relative change by looking at interactions between each of the smoothing methods with mean transition rate, proportion of multi‐behaviour sequences, total transitions, and number of activities. All variables were centered and scaled, and the intercept was removed to compare effects to no change in the control condition. Model including covariates had a lower AIC than the null model (no covariates).

Of the covariates, mean transitions per continuous sequence were found to have a statistically significant positive effect on relative change (*β* = 0.027, *p* < 0.05), whereas the number of activities had a significant negative effect (*β* = −0.029, *p* < 0.001). Duration smoothing methods had a negative interaction with the mean transitions such that, for the Duration smoothing, an increased number of transitions decreased performance (*β* = −0.031, *p* < 0.05). Similarly, the Bayesian smoothing method had a negative interaction with increasing rates of multi‐behaviour sequences such that the inclusion of more mixed sequences decreased performance gains (*β* = −0.093, *p* < 0.05).

**FIGURE 4:**
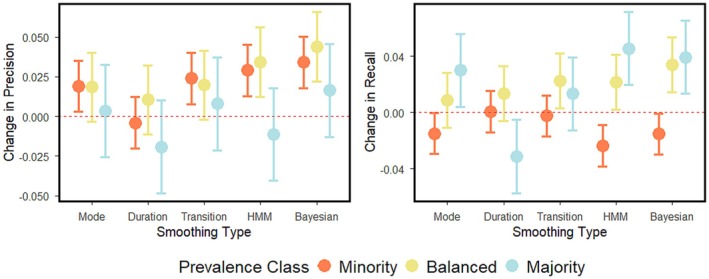
Change in performance (Precision, left, and Recall, right) for classes of behaviour prevalence (Minority, Balanced, and Majority classes) for each of the post‐processing methods averaged over random effects. Change is calculated relative to the control. The horizontal line represents no change (baseline control, no post‐processing). Points represent mean with error bars for 95% confidence intervals.

The effect of post‐processing on the minority classes was examined by comparing relative performance changes across *F*1‐score, Precision, and Recall depending on the class prevalence (Figure 4). For each performance metric, a linear mixed‐effects model assessed the effect of post‐processing method on classes of different prevalence with Species and cross‐validation replications included as random effects and no intercept (direct comparison with control condition). Smoothing method did not significantly affect precision across prevalence classes (Type III Wald *χ* ^2^ = 3.78, df = 8, *p* = 0.876). However, this interaction was strongly significant for recall (*χ* ^2^ = 36.65, df = 8, *p* < 0.001). Both Bayesian and HMM smoothing significantly increased recall in balanced (Bayesian: *β* = 0.034, *p* < 0.001, HMM: *β* = 0.021, *p* < 0.05) and majority classes (Bayesian: *β* = 0.039, *p* < 0.05, HMM: *β* = 0.04, *p* < 0.001) but decreased recall in minority classes (Bayesian: *β* = −0.024, *p* < 0.05, HMM: *β* = −0.015, *p* < 0.05). Similarly, mode‐based smoothing had a significant negative effect on the recall of minority classes (*β* = −0.015, *p* < 0.05) but a significant increase to recall of majority classes (*β* = 0.030, *p* < 0.05).

### 3.2 Ecological Sequence Case‐Study

The downstream effects of postprocessing decisions were demonstrated with a case study using the labelled as well as additional unlabelled data from deployment on free‐roaming individuals from the Sparkes_Koala dataset (Sparkes et al. 2025). The frequency and duration of both walking and foraging were compared (Table 3).

**TABLE 3:** Summarisation of behavioural events predicted from the sample of free‐roaming koala data with each of the post‐processing methods for two key behaviours.

Dataset	Labelled		Unlabelled	
Postprocessor	Mean frequency bout per individual	Mean duration bout per individual	Mean frequency bout per individual	Mean duration bout per individual
Behaviour: Locomotion				
TrueClass	1.75	110	Unknown	Unknown
None	5.67	19.6	12.9	4.79
Mode	6.75	39.4	6.17	21.2
Duration	5.83	25.8	12.9	4.79
Transition	7.25	28.9	7.83	11.2
HMM	5.5	25.2	6.12	4.58
Bayesian	1.5	134	1	26
Behaviour: Foraging				
TrueClass	1.75	175	Unknown	Unknown
None	7.2	12.3	683	12.8
Mode	3.78	28.2	208	50.5
Duration	4	26.3	369	23.1
Transition	4.11	24.6	378	22.8
HMM	5.89	13.5	210	164
Bayesian	2	76.8	28	407

*Note:* Frequency is expressed as a count, and duration in seconds. Table contains mean values across all individuals.

In the labelled data, there was a true mean frequency of 1.75 walks per individual per night with a mean duration of 110 s each. The base ML model predicted a mean frequency of 5.67 walks with a 19.6 s mean duration. Each of the postprocessors altered these predictions slightly differently, but the Bayesian model predicted a frequency (1.5 walks per individual) and mean duration (134 s) closest to the true values. In the unlabelled data, there were no true classes against which to compare, but the Bayesian models predicted fewer walking events than other methods. Similarly for foraging, in the labelled data, the Bayesian method was the closest approximation of true performance, and in the unlabelled data, Bayesian post‐processing resulted in fewest predicted bouts of feeding (ten‐fold fewer than other methods) at greater duration.

**FIGURE 5:**
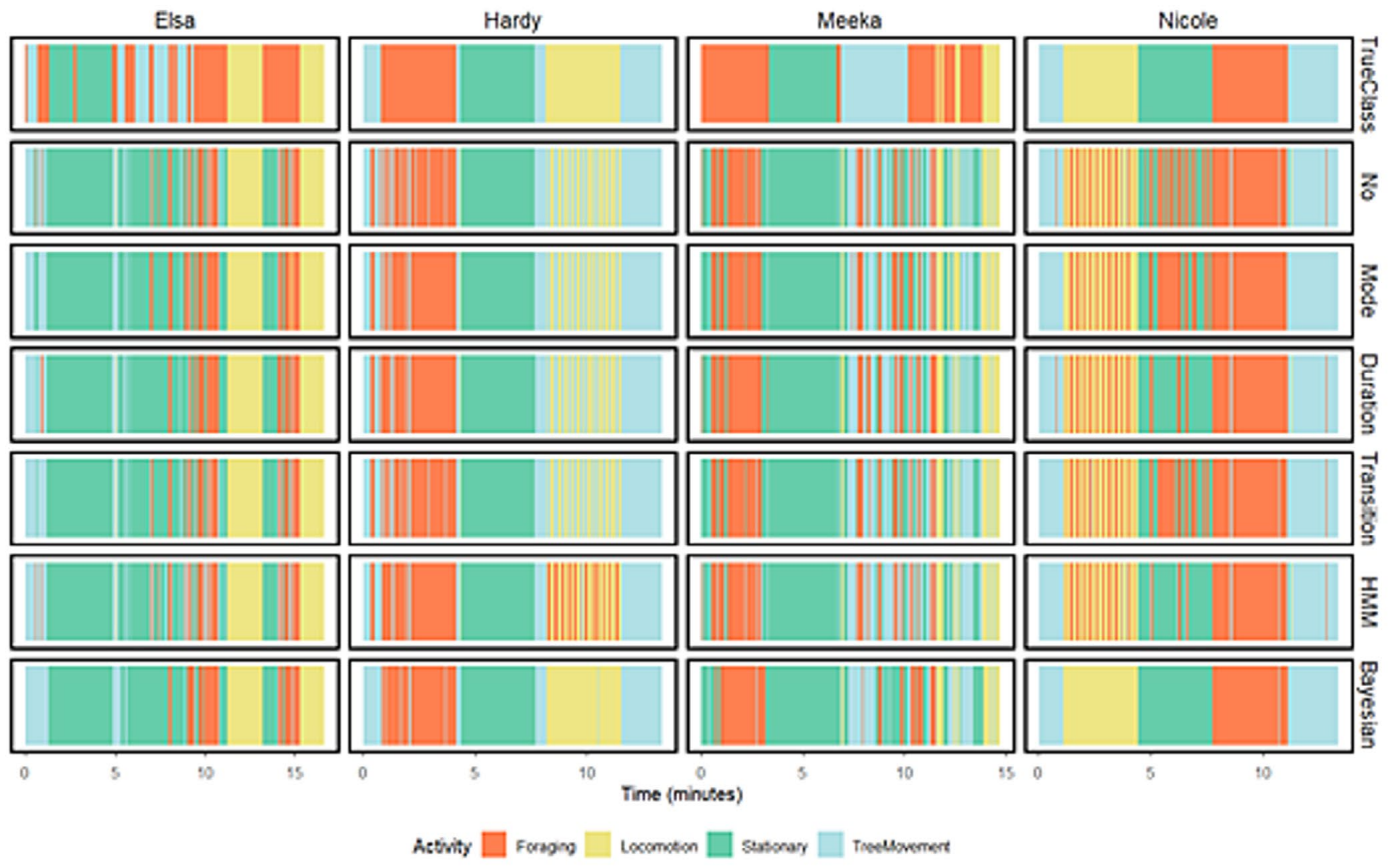
Change in predicted behavioural sequence for each individual and post‐processing method on the labelled Sparkes_Koala data for individuals with more than 5 min of labelled training data. Each column is an individual and each row represents a post‐processing smoothing method where ‘No’ is no smoothing and TrueClass are the original labels as applied by the researchers from synchronised videos. Each colour is a predicted activity (or true activity for True Class).

**FIGURE 6:**
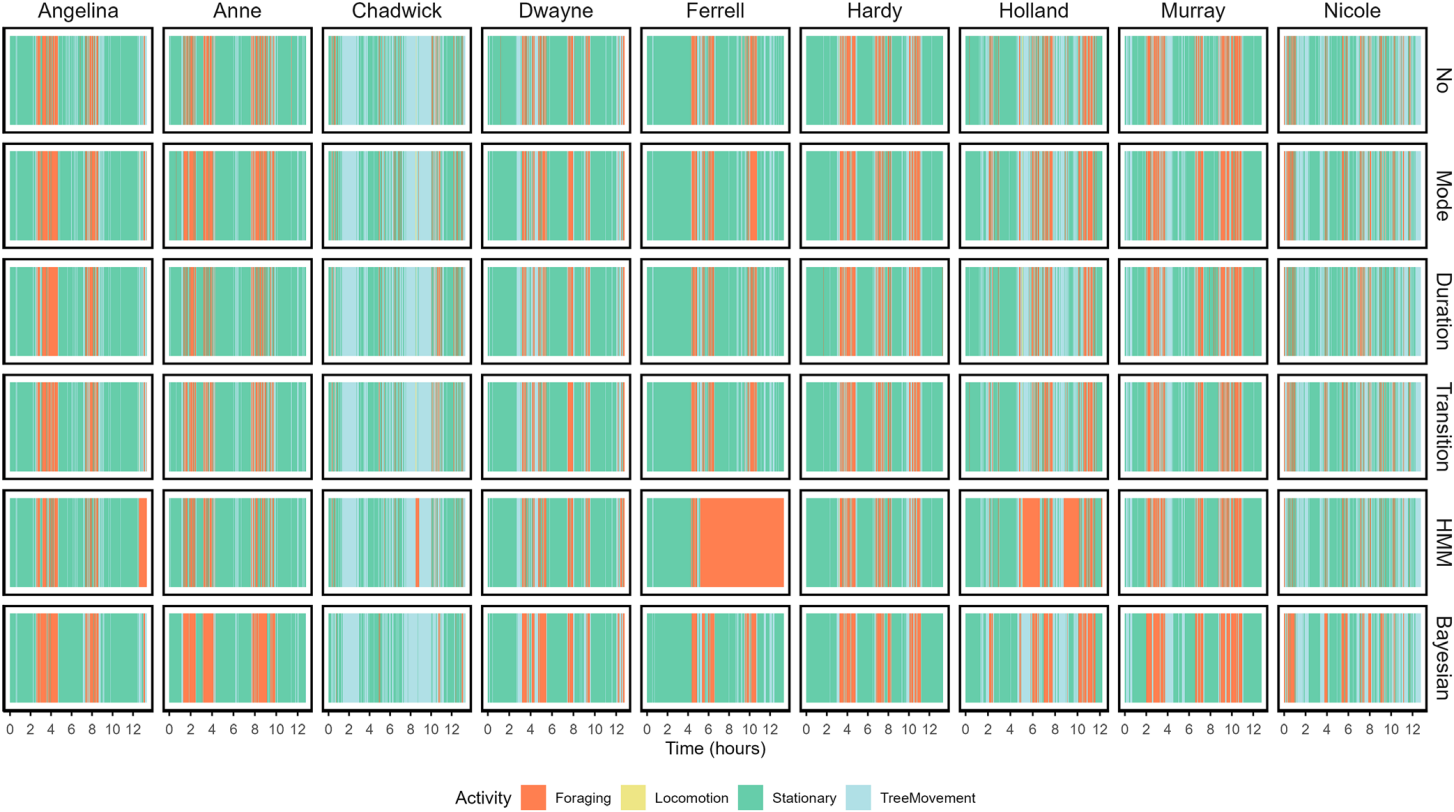
Change in predicted behavioural sequence for each individual and post‐processing method on the unlabelled Sparkes_Koala data for all individuals. Each column is an individual and each row represents a post‐processing smoothing method where ‘No’ is no smoothing. Each colour is a predicted activity.

The sequence of behaviours as predicted and smoothed by each method was also visualised for labelled (Figure 5) and unlabelled (Figure 6) Koala_Sparkes data.

## 4 Discussion

In this study, we compared the effect of five post‐processing techniques (Mode‐based, Duration‐based, Transition, HMM, and Bayesian) on the classification performance of accelerometer‐based behavioural classification predictions. The use of sequence‐informed post‐processors was hypothesised to smooth errors and improve performance without the collection of additional training data. Of the trialled post‐processors across 15 datasets, no single method achieved best performance on all trials. However, on average, Bayesian smoothing showed the greatest increase in performance, with an average relative performance gain of 6% across datasets and up to 20% on individual datasets, though this performance gain was closely followed by the HMM approach.

Performance of individual classes within each dataset depended on prevalence of the class within the dataset, with common classes (balanced prevalence or above) performing better than minority classes (less than balanced prevalence) in all cases. Analysis of precision (how many of the identified events were correct) and recall (how many of the total events were identified) showed that Bayesian and HMM smoothing methods biased performance towards detection of common classes, with statistically significant declines in the recall of minority classes. Although best‐performing on average, therefore, these methods would be inappropriate for research questions optimising for detection of the minority class.

Individual datasets also varied in their response to the post‐processors. This variance was partially explained by structure of the training dataset, with training datasets that contained more transitions per continuous sampling sequence generally resulting in greater performance gains in the post‐processing stage (presumably because there were more available transitions to learn from), but datasets with many possible behavioural classes benefiting less. This suggests that training data that includes natural transitions between behavioural states provides sequence context for the post‐processor to learn from, resulting in better‐informed smoothers, but, at high complexity (e.g., 10 classes), sequences became too difficult for these simple post‐processors to manage, and performance declines.

While the gains achieved with the post‐processing methods trialled in this research can appear trivial, importantly, they are comparable with gains seen across the broader wildlife animal accelerometry literature when working with similarly small datasets (i.e., excluding deep learning). Research comparing model architectures have reported accuracy differences of less than five‐percent between state‐of‐the‐art models (Yu et al. 2021; Otsuka et al. 2024; Bar‐Gera et al. 2025), and even custom‐designed solutions achieve performance improvements of less than 1 *F*1‐score point over baseline Random Forests (Ladds et al. 2017). The potential for Bayesian‐informed post‐processing to achieve an average gain of 6% improvement to the *F*1‐score at no additional training time, computational effort, or data acquisition, is therefore meaningful in context of the field.

### 4.1 Ecological Sequence Case‐Study

Our case study allows us to assess the downstream implications of analysing smoothed vs. raw behavioural predictions. We assessed a behavioural question typical of those asked in an applied accelerometry paper: what is the average frequency and duration of walking (minority class) and foraging (balanced class) bouts (continuous stretches of behaviour) across all koalas?

The original koala behaviour classification model had an estimated performance of 70% *F*1‐score and a 13% improvement (79% *F*1‐score) when post‐processed with the Bayesian smoother. In the labelled data, original predictions overestimated bout frequency while underestimating duration for both behaviours' bouts. For walking, unsmoothed predictions estimated three‐fold more walking events of five‐fold shorter duration than actually observed, and for foraging, four‐fold more feeding events of 14‐fold shorter duration than reality. Of the post‐processing methods, Bayesian smoothing achieved estimates closest to the true observations.

In the unlabelled deployment data, relative to unsmoothed and alternate methods, the Bayesian approach predicted both fewer and longer walking and foraging events. Without true class labels against which to compare in the unlabelled data, an accuracy assessment cannot be made. However, knowledge of the species should nevertheless allow the ecologist to assess whether an average of 12 walks of four seconds each and 683 foraging bouts of 12 s each (as per the unsmoothed base predictions) or one walk of 26 s and 28 foraging bouts of 6 min each (as per the Bayesian smoothed predictions) each night is a more appropriate reflection of koala ecology. While post‐processing may provide only modest improvements at the level of the behavioural budget (overall proportions of behaviour), it is, in fact, critical for the detailed insights available from bout analysis.

### 4.2 Recommendations for and Limitations of Application

Unlike many methodological studies that trial proposed methods on a single dataset (Wilson et al. 2026; Resheff et al. 2022; Grünewälder et al. 2012), by trialling these post‐processing methods across 15 diverse datasets, we are able to better approximate a generalisable insight. Based on performance improvements observed across these 15 datasets, together with finer‐scale evaluation of the case study dataset, we conclude that, of the trialled methods, the Bayesian post‐processor appears most suitable as a general starting point for trailing the use of a post‐processor for improving ML‐based behavioural classifications from accelerometer‐derived animal movement data. Selection of the post‐processor used in each study should, however, be carefully considered for each dataset and ecological context.

The field of accelerometer‐based animal behaviour classification has long been seeking a simplified analytical pipeline, yet there remains no standardised workflow for analysis. Rather than a failure of the field to converge on a solution, this lack of uniformity may actually reflect the genuine variety and diversity of the subject matter (Brown et al. 2013; Hoffman et al. 2024). Animals differ vastly in their behavioural profiles, and, equally, the questions we ask about their behaviours differ greatly between contexts such that there may not be, in fact, a one‐size‐fits‐all solution (e.g., Yu and Klaassen (2022) found benefit from a customised duration‐based smoother, which we did not find in our datasets, though we did find similar performance gains as previous research on HMMs [Grünewälder et al. 2012]). Rather, a match must be sought between the problem and the tool.

The limitations of each post‐processor must be considered. Models that perform well overall still make trade‐offs, such as sacrificing the recall of the minority class (i.e., underpredict rare classes, e.g., Bayesian and HMM models) or reducing the precision of the majority classes (i.e., over‐predict the majority class, e.g., Duration‐based smoothing). An apparent weakness of the Bayesian method in this study was declining performance in datasets with many (~10) behavioural classes, though this may be an artefact of many‐class models including more minority classes, which the Bayesian model underpredicts. The declining performance in these cases may be avoidable if each of the classes were similarly represented.

In some cases, it may be that no post‐processing approach will provide performance benefits. For example, a dataset with very high base‐classification performance (> 0.85 *F*1‐Score) may not require further refinement via post‐processing. Other datasets may simply see no benefit from smoothing. For example, the Vehkaoja_Dog dataset saw little benefit from any of the post‐processing methods such that even the best smoother did not show significant benefit on this dataset. Nor will post‐processing be able to account for completely incorrect predictions. If the original predictions from the machine learning classifier are very poor (such as an *F*1‐score of lower than 0.5), post‐processing will have a limited ability to resolve these errors.

Each post‐processing method considered in this paper presents strengths, weaknesses, and biases that will play off against the unique ecology and question of interest in each dataset, and it is the role of the ecologist to weigh up these options to select the most appropriate method. To assist with ecologist decision making, we have attempted to summarise our findings into usable recommendations (Table 4).

**TABLE 4:** Summarised recommendations for application of postprocessing to ecological data.

Method	Strength	Weakness	Application notes
Modal activity	Simple and deterministic. Removes isolated misclassifications within otherwise‐stable sequences	Suppresses probability of detecting rare and short‐duration behaviours. Ignores temporal structure beyond the window	Recommend for removing haphazard misclassifications from consistent behavioural bouts (e.g., long periods of uninterrupted sleep or grazing) when interested in average behavioural states rather than specifics. Avoid when short events are ecologically important
Duration of activities	Explicitly enforces minimum bout length	Empirically ineffective. Relies on arbitrary thresholds. Relies on representative training data examples	Not recommended. However, success has been found with a customised variant of this method in other studies (Yu and Klaassen 2022)
Transition between activities	Explicitly informed by ecology. Prevents impossible behavioural transitions	Dependent on training correctness and completeness of transitions in data. Requires training data containing sufficient examples of naturally transitioning behaviours	Recommended only when behaviour is bound by strong, well‐understood ecological sequence constraints (e.g., dive must be followed by return to surface, which must be followed by breathing [Patterson et al. 2019; Chimienti et al. 2016]). Transition‐probability weightings can be derived from the training data or explicitly defined from expert knowledge
Hidden Markov Model	Models temporal dependence explicitly. Most widely used in the literature	Sensitive to incomplete or incorrect transition and emission probabilities. Large variance in performance across datasets. Under‐predicts rare classes	Recommended for when behaviour is predictably sequential with consistent transition orders, and these sequences are adequately reflected in the training data. Not recommended for detection of minority classes
Bayesian	Greatest overall average performance improvements across all datasets	Performance declines in datasets with many (e.g., 10) behavioural classes. Under‐predicts rare classes	Recommended as a general‐purpose starting point for trialling post‐processing on new datasets. However, not recommended for detection of minority classes

### 4.3 Limitations and Future Directions

A limitation of all post‐processing methods that learn from sequences in the training data (in this case, the transition, HMM, and Bayesian methods) is the reliance on appropriate training data. These methods will present improvements only when the training samples contain sufficient and complete samples of the transitions that would be observed in the deployment data, with unnatural training data potentially leading to misleading estimates of natural durations and transitions (causing deterioration in the performance of the post‐processors). Notably, the Vehkaoja_Dog dataset—which saw no benefit from post‐processing—was the only dataset collected in which animals were instructed which behaviour to perform, in human‐proscribed sequences, suggesting that without natural sequences present in the training data, post‐processors potentially do not imbue benefit. Due to the challenges in continuous observation of wild animals, wild‐type training data is infrequent in the literature and most training data from wild animals is opportunistically collected for short, separated segments of observation. Even in captivity, natural sequences are not always observed (e.g., bellowing was rarely observed in captive koalas to be included and thus transitions to and from this behaviour could not be captured [Sparkes et al. 2025]). Indeed, the prevailing zeitgeist in the field has encouraged researchers to intentionally remove rare behaviours and transition windows from training datasets in the interest of improving performance of models. However, while including only single‐behaviour windows in the model development will result in better performance on said labelled set (if the test set has also been cleaned of transition windows), this does not inform us of the model's performance on the real, transition‐inclusive data that will be encountered in deployment (for detailed investigation see Resheff et al. (2024)). Furthermore, leaving out transition windows omits critical sequence information that could be used for post‐processing inference. Thus, we recommend, where possible, transition data between behavioural states be collected and retained when researchers are developing new training datasets, even if only for testing and post‐processing.

Our study represents a preliminary evaluation of multiple post‐processing approaches and aims to serve as a theoretical introduction to post‐processing rather than as an exhaustive exploration, and thus, there is much room for expansion. Several of the trialled post‐processing approaches required arguably arbitrary values (mode over a window of *five*, transition probability threshold of *twice*‐chance, etc.). In the interest of prioritising broad exploration over extensive depth, these threshold values were not systematically tuned, and thus, our results may not reflect these methods' optimal performance for each dataset. Exploration of alternative parameterisations could improve performance in these methods. Additionally, we generated our base classifications from only a single classifier, Random Forest; one of the most commonly implemented ML architectures in accelerometer‐based animal behaviour classification (Jeantet et al. 2026). As we were testing the performance of the post‐processors rather than initial classifications, we did not trial multiple base classifier types.

Future work would benefit from trialling the performance of sophisticated deep‐learning methods for post‐processing. For example, the Long Short‐Term Memory Model is a neural network that accounts for preceding classes when making future predictions (Otsuka et al. 2024) and can be scaled to manage more class categories than the presently trialled models if sufficient training data could be provided. While our work focused mainly on the transitions between behaviours and saw no benefit from the Duration method, incorporation of the duration of behaviours before they transition (as found beneficial in other studies e.g., Yu and Klaassen 2022) could further refine sequential analysis. Alternatively, hybrid and hierarchical smoothing strategies that combine elements of multiple methods may outperform any single approach. For example, a hierarchical system could be developed such that long periods of inactivity (as identified with a Vectorial Body Dynamic Acceleration (VeDBA; Wilson et al. 2019), commonly used as an activity metric) are aggressively smoothed with a wide‐window mode‐smoother but periods of activity, above the threshold, have a finer‐scale highly‐tuned smoother applied (noting the trade‐off between behavioural duration and smoothing window [Agarwal et al. 2026]).

Furthermore, while we have demonstrated the efficacy of sequence‐informed post‐processing in the accelerometry field, all sequential classification data could similarly benefit from such analysis. Most of the natural phenomena we study make sense only in the context of time and landscape. Breaking up our data into discontinuous chunks may be the easiest way to process and train our models, but when it comes to synthesising our findings and reintegrating the predictions for ecological interpretation, accounting for and leveraging the natural sequence and relationship of these results provides necessary, realistic context.

## 5 Conclusion

ML‐based behavioural classification represents only one stage in the pipeline from raw sensor data to ecological inference and while prior work has predominantly focused on fine tuning the models, our results demonstrate that significant performance gains can also be achieved downstream. Behaviour exists in a multi‐scale sequence hierarchy and accelerometer‐based behaviour classification may have much to benefit from greater integration of these temporal structures and higher‐order patterns to help draw meaningful ecological insights from our data. We recommend researchers interested in improving the performance of their behavioural classifications with post‐processing carefully consider the requirements of their ecological question, weighed up against the biases and trade‐offs of the possible post‐processors, to select a custom solution for their dataset.

## Author Contributions

**Oakleigh Wilson:** conceptualization (lead), data curation (lead), formal analysis (lead), investigation (lead), methodology (lead), visualization (lead), writing – original draft (lead), writing – review and editing (lead). **Hui Yu:** conceptualization (supporting), methodology (supporting), writing – review and editing (supporting). **David Schoeman:** formal analysis (supporting), methodology (supporting), supervision (lead), writing – review and editing (supporting). **Gabriella Sparkes:** data curation (supporting). **Christofer Clemente:** supervision (supporting), writing – review and editing (supporting).

## Funding

This work was supported by Ecological Society of Australia Incorporated (Student Research Grant, Ecological Society of Australia).

## Conflicts of Interest

The authors declare no conflicts of interest.

## Data Availability

All raw data was sourced from open‐source databases associated with the publications referenced in this manuscript. All code is available at https://github.com/OakAlice/PostProcessing. A single dataset example has been made available on the GitHub.
